# Angiogenic Potential of Various Oral Cavity–Derived Mesenchymal Stem Cells and Cell-Derived Secretome: A Systematic Review and Meta-Analysis

**DOI:** 10.1055/s-0043-1776315

**Published:** 2023-11-23

**Authors:** Madhura Shekatkar, Supriya Kheur, Shantanu Deshpande, Avinash Sanap, Avinash Kharat, Shivani Navalakha, Archana Gupta, Mohit Kheur, Ramesh Bhonde, Yash P. Merchant

**Affiliations:** 1 Department of Oral Pathology and Microbiology, Dr. D. Y. Patil Dental College and Hospital, Dr. D. Y. Patil Vidyapeeth, Pimpri, Pune, India; 2 Department of Pediatric and Preventive Dentistry, Bharati Vidyapeeth (Deemed to be) University Dental College and Hospital, Navi Mumbai, India; 3 Regenerative Medicine Laboratory, Dr. D. Y. Patil Dental College and Hospital, Dr. D. Y. Patil Vidyapeeth, Pimpri, Pune, India; 4 Department of Oral Pathology and Microbiology, Dr. D. Y. Patil Vidyapeeth, Pimpri, Pune, India; 5 Department of Prosthodontics, M.A. Rangoonwala College of Dental Sciences and Research Centre, Pune, India; 6 Dr. D. Y. Patil Vidyapeeth, Pimpri, Pune, India; 7 Department of Oral and Maxillofacial Surgery, Dr. D. Y. Patil Dental College, and Hospital, Dr. D. Y. Patil Vidyapeeth, Pimpri, Pune, India

**Keywords:** secretome, angiogenesis, angiogenic growth factors, cytokine analysis

## Abstract

Recent evidence suggests the immense potential of human mesenchymal stem cell (hMSC) secretome conditioned medium-mediated augmentation of angiogenesis. However, angiogenesis potential varies from source and origin. The hMSCs derived from the oral cavity share an exceptional quality due to their origin from a hypoxic environment. Our systematic review aimed to compare the mesenchymal stem cells (MSCs) derived from various oral cavity sources and cell-derived secretomes, and evaluate their angiogenic potential. A literature search was conducted using PubMed and Scopus from January 2000 to September 2020. Source-wise outcomes were systematically analyzed using *in vitro* , *in vivo* , and *in ovo* studies, emphasizing endothelial cell migration, tube formation, and blood vessel formation. Ninety-four studies were included in the systematic review, out of which 4 studies were subsequently included in the meta-analysis. Prominent growth factors and other bioactive components implicated in improving angiogenesis were included in the respective studies. The findings suggest that oral tissues are a rich source of hMSCs. The meta-analysis revealed a positive correlation between dental pulp–derived MSCs (DPMSCs) and stem cells derived from apical papilla (SCAP) compared to human umbilical cord–derived endothelial cell lines as a control. It shows a statistically significant positive correlation between the co-culture of human umbilical vein endothelial cells (HUVECs) and DPMSCs with tubule length formation and total branching points. Our meta-analysis revealed that oral-derived MSCs (dental pulp stem cells and SCAP) carry a better angiogenic potential *in vitro* than endothelial cell lines alone. The reviewed literature illustrates that oral cavity–derived MSCs (OC-MSCs) increased angiogenesis. The present literature reveals a dearth of investigations involving sources other than dental pulp. Even though OC-MSCs have revealed more significant potential than other MSCs, more comprehensive, target-oriented interinstitutional prospective studies are warranted to determine whether oral cavity–derived stem cells are the most excellent sources of significant angiogenic potential.

## Introduction

Oral cavity–derived dental pulp stem cells (DPSCs) have gained attention due to their potential use in regenerative medicine. These stem cells are known for their unique characteristics that make them distinct from other stem cell sources. Some exceptional criteria of oral cavity–derived DPSCs are their mesenchymal stem cell (MSC) characteristics, ease of accessibility, multilineage differentiation, regenerative capacity with high angiogenic potential, and immunomodulatory properties with low immunogenicity. Despite their potential advantages, using oral cavity–derived stem cells for oral cancer treatment and reconstruction poses several challenges. Oral cancer creates a hostile tumor microenvironment characterized by inflammation, hypoxia, and immune suppression. Stem cells may face difficulty surviving and exerting their regenerative properties in such an environment. There is a risk that the harvested stem cell population could be contaminated with cancer cells, which can lead to cancer recurrence if transplanted back into the patient. Moreover, oral carcinoma contains a population of neoplastic cells with aggressive stem cells that are difficult to distinguish from healthy cells. Angiogenesis or neovascularization is a dynamic process involving new blood vessels that form from existing blood vessels. 1 Oral cavity stem cells secrete various angiogenic factors, including vascular endothelial growth factor (VEGF), fibroblast growth factor (FGF), platelet-derived growth factor (PDGF), and others. These factors attract endothelial cells and support the formation of new capillaries.

Oral cavity stem cells can trigger angiogenesis when introduced into a tissue requiring regeneration or healing. It is crucial during development, along with various physiological and pathological processes. 2 Angiogenesis occurs lifelong, starting in the uterus and continuing into old age. Furthermore, capillaries are required to exchange nutrients and metabolites in all tissues. 3 Angiogenesis is paramount concerning wound healing due to its critical role in growing a new capillary network from the granulation tissue, which plays a pivotal role in chronic inflammation. 3 Wound healing is a complex procedure involving overlapping events, including inflammatory, proliferative, and remodeling phases. Many growth factors and cytokines participate in the proliferative phase, of which angiogenic growth factors hold a prime role. 4 Revascularization is regulated by a complex interaction between various growth factors, including but not limited to VEGF, FGF, angiopoietins (ANG), PDGF, transforming growth factor-α (TGF-α), and transforming growth factor-β (TGF-β). 5 Each factor plays a separate role in inducing, initiating, and amplifying cell proliferation, cell migration, stabilization, wound healing, inflammation, and suppression of angiogenesis. 1 6 Several growth factors like VEGF, FGF-2, and PDGF have been used clinically to augment angiogenesis for various therapeutic applications. However, lack of spatiotemporal control over the release of these proangiogenic proteins has led to numerous complications, including leaky vasculature. Cell-based therapies are evolving therapeutic options for deranged angiogenesis. 7

MSCs derived from human placental tissue, bone marrow, or umbilical cord tissues provide a novel strategy for the induction of angiogenesis. Various studies have demonstrated the ability of MSCs to differentiate into endothelial cells and provide vascular stability. In addition, MSCs secrete an extended milieu of growth factors, cytokines, extracellular vesicles (EVs), and messenger ribonucleic acids (mRNAs) implicated in a wide range of biological processes. Interestingly, “secret factors” (secretomes) from MSCs promote angiogenesis and amend wound healing in virtue of potent paracrine signaling, yielding proangiogenic factors. 5 Although hMSCs isolated from various sources have exhibited proangiogenic potential, knowledge about the ideal source (cells or secretomes, source-wise potential, and ease of sample collection) remains obscure. Oral tissues originate from mesenchymal and ectodermal germ layers that add to their value, making them the ideal source for isolation and therapeutic applications. Stem cells are influenced by their *in vivo* environment, which projects through their therapeutic properties. 8 The stem cell niche includes cellular and extracellular matrix components, tissue location, innervation, and blood supply. The oral cavity is highly vascularized and yields better-quality stem cells with potent angiogenic potential. Rapid wound healing in the oral cavity can explain its unique potential. Their high proliferation and unique secretory profile can be attributed to their hypoxic condition. Oral cavity–derived cells are multipotent; primitive oral tissues such as dental follicles harbor oral cavity–derived MSCs (OC-MSCs). Therefore, MSCs isolated from various sources from the oral cavity comprise a powerful weapon to battle numerous diseases. 9

In recent decades, stem cell proliferation from various adult tissues has been a provoking tool in advanced sciences. Previous studies have revealed the role of dental pulp–derived MSCs (DPMSCs) and stem cells from human exfoliated deciduous teeth (SHED) in enhancing the cascade of angiogenesis. Our systematic review aimed to compare OC-MSCs and cell-derived secretomes and evaluate their angiogenic potential. The subsequent meta-analysis with compatible data analyses whether OC-MSCs (DPSC and stem cells derived from apical papilla [SCAP]) carry a better angiogenic potential *in vitro* than endothelial cell lines alone. Extensive collaborative research is required to conclude which oral-derived stem cells have the best angiogenic potential. This systematic review focuses on the potential of MSCs and their secretomes derived from various oral tissues such as gingival tissue, dental pulp, periodontal ligament (PDL), mandibular bone, and buccal fat, with particular emphasis on angiogenesis.

## Methods

This study was conducted according to the Preferred Reporting Items for Systematic Reviews and Meta-Analyses (PRISMA) guidelines. The research proposal has been registered in PROSPERO (registration no: CRD42021282497).

Based on the PRISMA criteria, the research question for this review was framed in the PICO format as the following: Which is the best oral source of MSCs for augmenting angiogenesis at the implanted site?

The terms used to identify studies based on the elements of the PICO format were as follows:

- Population: i *n vitro* studies, *in vivo* studies, and ex vivo studies.
- Intervention: OC-MSCs.
- Comparison: between various OC-MSCs.
- Outcome: angiogenesis at the desired site of implantation.

The inclusion criteria of the study were the following:

- Articles published in the English language.
- Studies relevant to the topic published from January 1, 2000 to March 2023.
- Studies showing *in vitro* , *in vivo* , and *in ovo* results for angiogenesis of OC-MSCs.
- Studies having well-defined information regarding the angiogenic potential of OC-MSCs.

The exclusion criteria of the review included the following:

- Abstracts.
- Reviews.
- Letter to the editor.
- Editorials.
- Case reports.
- Short communication.
- Commentaries.
- Articles in languages other than English.

Systematic computer searches were performed on two electronic databases: PubMed and Scopus. The following keyword combinations were used to search articles:

- “Dental stem cells AND Angiogenesis AND conditioned media.”
- “Dental stem cells AND Angiogenic potential AND conditioned media.”
- “Dental stem cells AND Angiogenesis.”
- “Dental stem cells AND Angiogenic potential.”

Along with the electronic search, a hand search was also performed to find the missed articles. Articles published between January 1, 2000 and March 1, 2023 were included in the survey. Two reviewers (M.S. and S.K.) independently evaluated the titles and abstracts of the retrieved publications pertaining to the covered research topic during the initial screening. If material relevant to the inclusion criteria was provided in the abstract, or if the title was relevant but the abstract was unavailable, a full-text report was acquired. The complete text of the articles was then screened to find those that matched the inclusion criteria. If the work appeared to meet the inclusion criteria, the authors were contacted to seek further information. Articles with full-text reports only were evaluated in this systematic review. Studies that only published abstracts were removed because evidence revealed differences between data given in abstracts and those supplied in the final published complete report. Two review authors (M.S. and S.K.) separately collected data using a specifically designed data extraction sheet ( Table 1 ). A third (S.D.) and a fourth (Y.M.) reviewer handled any disagreements about the inclusion of publications or data extraction.

**Table 1:** Tabular representation of qualitative data obtained from literature search for included studies

Sl. no.	Study	Source of stem cells	Type of study	Model used	Factors assessed for angiogenesis	Method used for analysis	Use of stem cells/conditioned media	Use of preconditioning (yes/no)	Use of co-culture with MSCs (yes/no)	Results obtained
**Dental pulp–derived mesenchymal stem cells (DPMSCs)**										
1.	Li et al 10	Dental pulp	*In vivo*	Mice	VEGFR1, VEGFR2, VE-cadherin, ETV2, and CD31	Real-time polymerase chain reaction (RT-PCR)	Cells	ETV2 transfected	Human umbilical vein endothelial cell (HUVEC)	Dental pulp stem cell (DPSCs) proved as potential candidates for clinical applications in therapeutic tissue engineering
2.	Boreak et al 11	Dental pulp	In ovo	Yolk sac membrane (YSM)	VEGFA, FGF-2, CXCL8, VEGF, and angiopoietin-2	Enzymelinked immunosorbent assay (ELISA) and RT-PCR	Conditioned media	Metformin, cisplatin (negative control) L-arginine (positive control)	No	Metformin treated conditioned media derived from DPSCs enhanced the level of angiogenic activity in the YSM
3.	Li et al 12	Dental pulp	*In vivo*	Rats	Angiogenin, basic fibroblast growth factor (bFGF), hepatocyte growth factor (HGF), HIF-1α, interleukin-8 (IL-8), monocyte chemotactic protein 1 (MCP-1), platelet-derived growth factor (PDGF), and vascular endothelial growth factor (VEGF)	RT-PCR	Cells	No	No	Stem cells from the dental pulp provided greater therapeutic effects compared to stem cells from the umbilical cord
4.	Li et al 13	Dental pulp	*In vivo*	Rats	VEGF, VEGFR-2 (Flk1)	RT-PCR and ELISA	Cells	Nell-1	HUVEC	Nell-1 could promote endothelial vessel formation and enhance the angiogenic factor expression when treated over the DPSCs or HUVECs
5.	Alghutaimel et al 14	Dental pulp	*In vivo*	Mice	VEGF-A, FGF-2	RT-PCR	Cells	Decellularized dental pulp (DDP) matrix of bovine origin treated the DPSCs	No	DDP seeded along with the DPSCs provided greater angiogenic efficiency that singularly seeded the DDP
6.	Zhou et al 15	Dental pulp	*In vitro*	HUVECs	VEGF	RT-PCR	Cells	Transfection of miR-378a. hedgehog/Gli1 signaling inhibition	HUVECs	Extracellular vesicles derived from the DPSC transfected with miR-378a could enhance angiogenic proliferation *in vitro*
7.	Huang et al 16	Dental pulp	*In vitro*	HUVECs	VEGF and kinase-insert domain-containing receptor (KDR)	RT-PCR	Cells	Lipopolysaccharide (LPS)	HUVECs	Inflammatory stimulation
8.	Afami et al 17	Dental pulp	*In vitro*	Microbes	Angiogenin, EGF, FGF, PDGF, INF-gamma, VEGF, insulinlike growth factor (IGF), and angiopoietin	Heat map	Both	(Naphthalene-2-ly)-acetyl-diphenylalanine-dilysine-OH (NapFFεKεK-OH)	Hydrogel	Increased vascular components
9.	Liao et al 18	Dental pulp	*In vivo*	Mice	VEGF and AngII	qRT-PCR analysis and immunofluorescence staining	Cells	No	No	Enhanced wound healing
10.	He et al 19	Dental pulp	*In vitro*	C. albicans biofilms	Hyphal wall protein1 (hwp1), agglutininlike sequence protein 3 (als3) and cell surface hydrophobicity (csh1)	RT-PCR	Cells	Norspermidine (NSPD)	GelMA hydrogels	NSPD did not directly influence the angiogenic properties of the DPSCs
11.	Guo et al 20	Dental pulp	*In vivo*	Rat	Angiogenin, EGF, bFGF, and HGF	Not mentioned	Cells	No	Human adipose microvascular endothelial cells (HAMECs)	Co-culture of the DPSC with the HAMECs yielded denser vascular bundles compared to endothelial cells alone
12.	Luzuriaga et al 21	Dental pulp	*In vitro*	Mouse liver sinusoidal endothelial cells (mLSECs)	VEGF	Flow cytometry	Cells	No	No	Use of the DPSC-enhanced prevascularized engraftments improves cell–graft integration compared to nonvascularized grafts
13.	Merckx et al 22	Dental pulp	In ovo	Chorioallantoic membrane (CAM) of chicken embryos	Angiogenin, angiopoietin-1 (Angpt-1), HGF, insulinlike growth factor-binding proteins (IGFBPs), monocyte chemoattractant protein-1 (MCP-1), urokinase plasminogen activator (uPA), and VEGF	Transmission electron microscopy, high-resolution flow cytometry, and ELISA	Both	No	Co-culture with bone marrow–derived MSCs (BM-MSCs)	Positive paracrine effects on endothelial cell migration and *in ovo* blood vessel formation, with a stronger potential for BM-MSCs was found
14.	Caseiro et al 23	Dental pulp	*In vivo*	Rats	Angiopoietin-2 (Ang), EGF, endothelin-1 (EDN1), fibroblast growth factor 1 and 2 (FGF-1 and FGF-2), PDGF-AA and PDGF-AB/BB, transforming growth factor alpha (TGFα), transforming growth factor beta 1, 2, and 3 (TGF-β1, -2, and -3), tumor necrosis factor alpha (TNFα), TNFβ, VEGF-A, VEGF-C, and VEGF-D	PCR	Both	No	Co-culturing was done with umbilical cord–derived MSCs	UC-MSCs provide a wider variety and greater concentration of relevant growth factors and cytokines
15.	Makino et al 24	Dental pulp	*In vivo*	Rats	TNF-α, VEGF, and bFGF	Immunohistological staining	Both	No	No	Increased capillary formation achieved
16.	Chen et al 25	Dental pulp	*In vitro*	Endothelial cell line	VEGF, PDGF, SDF-1, and GAPDH	IHC and PCR	Cells	No	No	VEGF expression was higher in pulp tissue from teeth with deep caries (cDPMSCs) than in normal tissue
17.	Li et al 26	Dental pulp	*In vitro*	Endothelial cell line	KDR and CD31	Immunofluorescence analysis and RT-PCR	Cells	No	Human decellularized dental pulp matrix (hDDPM)	Increased proliferation of blood vessel-like structures was evident
18.	Wang et al 27	Dental pulp	*In vitro*	Endothelial cell line	VEGF	ELISA, two-photon laser microscopy	Cells	No	HUVECs	Extracellular vesicles from the DPMSCs can promote angiogenesis in an injectable hydrogel *in vitro*
19.	Zhou and Sun 28	Dental pulp	*In vitro*	Endothelial cell line under hypoxic conditions	VEGF, FGF, vWF, VEGFR2, VE-cad, HIF-1α, and CD31	PCR	Cells	No	No	Hypoxic conditions enhanced the tube formation of the DPMSCs *in vitro*
20.	Qu et al 29	Dental pulp	*In vitro*	HUVECs	Angiopoietin-1, VEGFA, and ribosomal protein L13a (RPL13a)	PCR and ELISA	Both	No	No	DPMSCs derived from conditioned medium (CM) could enhance capillary tube formation
21.	Zhu et al 30	Dental pulp	*In vivo*	Mice	VEGF and SDF-1α	PCR and ELISA	Both	No	No	Enhanced expression of VEGF and SDF-1α was observed
22.	Li et al 31	Dental pulp	*In vitro*	Endothelial cell line	VEGF, FGF, ANG-1, and PDGFA	RT-PCR and immunofluorescence	Cells	IGFBP5	No	IGFBP5 overexpression enhanced the expressions of angiogenic differentiation markers
23.	Lu et al 32	Dental pulp	*In vitro*	Endothelial cell line	p-AKT and cyclin D1	RT-PCR and Western blotting	Cells	VEGF and IGF-1	No	Combined treatment with VEGF and IGF-1 provided a synergistic effect on the angiogenic potential of DPMSCs derived from carious teeth
24.	Youssef et al 33	Dental pulp	*In vitro*	Endothelial cell line	VEGF	PCR, flow cytometry	Cells	Mineral trioxide aggregate (MTA), calcium hydroxide (Ca [OH]2, Biodentine (BD) and Emdogain	No	The treatment of MTA-enhanced VEGF expression, Ca (OH)2, BD, and Emdogain
25.	Rapino et al 34	Dental pulp	*In vitro*	Endothelial cell line	EDN1, VEGF, IL–6, and PGE2	ELISA	Cells	Chitlac-coated BisGMA/triethylene glycol dimethacrylate (TEGDMA) methacrylic thermosets	No	The addition of Chitlac-coated BisGMA/TEGDMA methacrylic thermosets resulted in tubules with an increased diameter and improved the differentiation of angiogenic cell types
26.	Dubey et al 35	Dental pulp	*In vitro*	Endothelial cell line	VEGF	Light and fluorescence microscopy	Cells	Clindamycin (CLIN) and minocycline (MINO)	HUVECs	There was enhanced cell proliferation and capillary tube formation
27.	Delle Monache et al 36	Dental pulp	*In vitro*	Endothelial cell line	FGF, VEGF, and EGF	Immunofluorescence, and Western blotting	Cells	Complete endothelial medium 2 (EGM-2)	HUVECs	EGM-2-treated DPMSCs formed tubelike structures that were more stabilized compared to HUVECs alone
28.	Gong et al 37	Dental pulp	*In vitro*	Endothelial cell line	VEGF	Immunofluorescence microscopy, PCR, and ELISA	Cells	EphrinB2-Fc or EphB4-Fc	HUVECs	EphrinB2-Fc or EphB4-Fc enhanced the DPMSCs to form blood vessels with increased secretion of VEGF
29.	Schertl et al 38	Dental pulp	*In vitro*	Endothelial cell line	PECAM1, VEGF-A, and KDR	Flow cytometry, and qRT-PCR analysis	Cells	TEGDMA	No	Treatment with 0.25 mM of TEGDMA downregulated angiogenic factor expression, while at 0.1 mM concentration angiogenesis was not affected
30.	Luzuriaga et al 39	Dental pulp	*In vivo*	Mouse	VEGF	PCR, flow cytometry, and Western blotting	Cells	No	No	Dental pulp–derived cells contributed to the generation of neovasculature in brain tissue
31.	Zou et al 40	Dental pulp	*In vitro*	Endothelial cell line	VEGF, HIF-1α, ANG1, and ANGPTL4	ELISA	Cells	Sema 4D/plexin B1	No	Signaling through sema 4D/plexin B1-induced endothelial differentiation of the DPMSCs
32.	Bindal et al 6	Dental pulp	*In vitro*	Endothelial cell line	FGF, VEGF-A, HGF, PDGF-BB, MCP-1, and CCL5	RT-qPCR array	Cells	LPS, human platelet lysate (HPL), platelet-rich plasma	No	20% HPL has been shown to provide the most optimal environment to induce proangiogenic factors in inflammatory DPMSCs
33.	Jin et al 41	Dental pulp	*In vitro*	Endothelial cell line	VEGF, FGF, PDGF, TGF-β	RT-PCR and immunofluorescence	Cells	Concentrated growth factor (CGF) scaffold	HUVECs	At low doses, CGF could potentially stimulate endothelial cell proliferation and migration
34.	Gharaei et al 42	Dental pulp	*In vitro*	HUVEC line	VEGF, IGF-1, SDF-1, IGFBP-2,3, MMP-9, TIMP-1, and Ang-1	ELISA, RT-PCR, and protein profiling array	Both	No	No	CM released from hDPMSCs can trigger pronounced angiogenic effects
35.	Dou et al 43	Dental pulp	*In vitro*	Endothelial cell line	VEGFA, HIF-1A, KDR(VEGFR2), TGFβ1, BMP-2, bFGF, HGF, TNF-α, Runx-2, and Notch-1	PCR, flow cytometry, and ELISA	Cells	Hypoxic conditions	No	Hypoxia could promote angiogenesis of the DPMSCs graft via the HIF-1ɑ signaling pathway
36.	Aksel et al 44	Dental pulp	*In vitro*	Endothelial cell line	VEGF	ELISA and PCR	Cells	Fibrin gel integrated demineralized dentin matrix	No	Increased angiogenic marker expression
37.	Lambrichts et al 45	Dental pulp	*In ovo* and *in vivo*	Chorioallantoic membrane, mice	VEGF, angiogenin, dipeptidyl peptidase IV, angiopoietin-1, EDN1, IGFBP-3, IL-8, urokinase-type plasminogen activator, MCP-1	Histopathologic staining	Both	No	No	hDPMSCs significantly augmented blood vessel growth in this ovo model for angiogenesis; also, pulp vascularization was obtained in a transplanted scaffold in the immune-compromised mice model
38.	Silva et al 46	Dental pulp	*In vivo*	Mice	VEGF, VEGFR2, and IL-8	ELISA	Cells	Lipoprotein receptor–related protein 6 (LRP6) and Frizzled6, recombinant human Wnt1 (rhWnt1), and recombinant human VEGF165 (rhVEGF165)	No	Lipoprotein receptor–related protein 6 silenced DPMSCs downregulated VEGF expression also showed fewer blood vessel formation in the mice model
39.	Aksel and Huang 47	Dental pulp	*In vitro*	Endothelial cell line	von Willebrand factor (vWF)	Immunofluorescence	Cells	Endothelial growth medium-2 (EGM-2)	No	EGM-2-induced cells showed improved vessel formation compared to noninduced cells
40.	Zou et al 48	Dental pulp	*In vitro*	Endothelial cell line	VEGF	ELISA and PCR	Cells	Sema4D/plexin B1	HUVECs	Sema4D/plexinB1 signaling exerts profound effects on enhancing VEGF secretion and angiogenesis
41.	Nam et al 49	Dental pulp	*In vivo*	Mice	VEGF, α-smooth muscle actin (α-SMA), PDGF receptor β (PDGFRβ), and CD146	Immunofluorescent staining	Cells	No	HUVECs	
42.	Lee et al 50	Dental pulp	*In vitro*	HUVECs	VEGF, FGF-2, VEGFRs, PECAM-1, and VE-cadherin	PCR	Both	Nanocomposite cements	No	The conditioning with nanocomposite cements-hDPMSC-CM showed the highest tubular number of HUVECs
43.	Lee et al 51	Dental pulp	*In vitro*	HUVECs	VEGF, PDGF, FGF-2, platelet endothelial cell adhesion molecule 1 (PECAM-1), and VE-cadherin	PCR	Both	Baicalein	No	Baicalein conditioning increased capillarylike tube formation significantly
44.	Spina et al 52	Dental pulp	*In vitro*	Collagen scaffolds	VEGF and PDGFA	PCR and IHC	Cells	New Zealand Foetal Bovine Serum	No	Expression of *VEGF* and *PDGFA* . hDPMSCs cultured in NZ-FBS were found to produce higher mRNA levels of the said angiogenic factors
45.	Kuang et al 53	Dental pulp	*In vivo*	Mice	VEGF and HIF-1α	PCR	Cells	Hypoxic conditions	No	After 4 weeks, the hypoxia group significantly enhanced angiogenesis inside the pulp chamber
46.	Shen et al 54	Dental pulp	*In vivo*	Mice	VEGF, SDF-1, MCP-1, PDGF-BB, IGF-1, TGF-β, and bFGF	IHC, laser Doppler flowmetry	Both	No	No	DP-CM was shown to significantly improve the recovery of persistent blood flow in the ischemic hindlimb of mice
47.	Dissanayaka et al 55	Dental pulp	*In vivo*	Mice	VEGF	ELISA	Cells	No	HUVECs	The extracellular matrix produced by the DPMSCs promoted the stabilization and remodeling of capillarylike structures formed by the HUVECs
48.	Boyle et al 56	Dental pulp	*In vitro*	HUVECs	VEGF	PCR, flow cytometry	Cells	TNF alpha	No	TNF alpha increased the angiogenesis of DPMSCs
49.	Liu et al 57	Dental pulp	*In vitro*	HUVECs	VEGF, kinase insert domain receptor (KDR), and FGF	Western blotting and RT-PCR	Lentiviral vector-transfected cells	MiR-424	No	Inhibition of miR-424 function promoted endothelial cell differentiation of hDPMSCs, whereas miR-424 overexpression inhibited their angiogenic potential
50.	Bronckaers et al 58	Dental pulp	*In ovo*	Human microvascular endothelial cell line 1 (HMEC-1), chicken chorioallantoic membrane, mouse brain endothelial cells (MBECs)	VEGF, IL-8, MCP-1, and FGF-2	ELISA and RT-PCR	Both	No	No	An increased number of capillary formations was evident
51.	Janebodin et al 59	Dental pulp	*In vivo*	Mice	VEGF	PCR	Cells	No	BM-MSCs	DPMSCs' ability to induce vessel formation was more efficient than BMSCs
52.	Ishizaka et al 60	Dental pulp	*In vivo*	Mice	Granulocyte monocyte colony-stimulating factor (GM-CSF), matrix metalloproteinase-3 (MMP-3), and VEGF-A	Flow cytometry	Both	No	Bone marrow, adipose tissue MSCs	DPMSCs have more significant potential for angiogenesis
53.	Dissanayaka et al 61	Dental pulp	*In vitro*	HUVECs	CD117, VEGF, CD34, and Flk-1	PCR	Cells	No	Endothelial cells	Matrigel assay showed that the addition of DPMSCs stabilized preexisting vessel-like structures formed by endothelial cells and increased their longevity
54.	Iohara et al 62	Dental pulp	*In vivo*	Mice	VEGF, MMP, CSF, CXCR4, and SDF1/CXCL12	PCR	Cells	No	No	It improved limb ischemia in the hindlimb of the mice model
**Stem cells from human exfoliated deciduous teeth (SHED)**										
1.	Wu et al 63	SHED	*In vivo*	Mice	VEGFA, PDGFA, and angiopoietin	RT-PCR	Cells	No	HUVEC and SHED exosomes	SHED exosomes provide expanded possibilities to enhance angiogenesis and pulp regeneration
2.	Han et al 64	SHED	*In vivo*	Mice	VEGF	ELISA	Both	Transfection of premade siRNA for HIF-1 alpha signal silencing	HUVECs	HIF-1 alpha signaling along with VEGF has a potent role for the use of SHED in regenerative medicine
3.	Zaw et al 65	SHED	*In vitro*	HUVECs	Bcl-2, NF-κB1, VEGFA, CXCL8, and CXCR1	ELISA, PCR, and flow cytometry	Cells	NF-κB decoy oligodeoxynucleotides (ODNs) or scramble (control)	Human dermal microvascular endothelial cells (HDMECs)	Increased expression of angiogenic factors was observed with co-culture
4.	Atlas et al 66	SHED	*In vivo*	Mice	VEGF, HGF, and PDGF-BB	Not mentioned	Cells	No	Endothelial cells	SHED takes part in the prevascularization process to further cause maturation of the vasculature
5.	Guo et al 67	SHED	*In vivo*	Minipigs	HIF-1a and VEGF	RT-PCR	Cells	No	Regenerated dental pulp stem cells and SHED together (R-SHED), HUVEC	The tube forming parameters on a Matrigel showed highest results for R-SHED. Likewise, the expression of angiogenic markers were higher in R-SHED group compared to the controls
6.	Wang et al 4	SHED	*In vitro*	HUVECs	VEGF, VEGFR2 CD31 and DLL4	PCR and ELISA	Cells	Treatment with shear stress.	No	Shear stress–induced arterial endothelial differentiation of SHED and VEGF-DLL4/Notch-EphrinB2 signaling was involved in this process
7.	Gong et al 68	SHED	*In vitro*	HUVECs	VEGF, FGF beta, and hEGF	IHC and PCR	Cells	No	HUVECs and decellularized matrix	Endothelial-induced SHED provided better angiogenesis
8.	Kim et al 69	SHED	*In vivo*	Mice		PCR and IHC	Cells	No	HUVECs	Co-culture of HUVECs and SHED could provide enhanced angiogenesis *in vivo*
9.	Gorin et al 70	SHED	*In vivo*	Mice	VEGF, FGF-2, HGF	Flow cytometry ELISA, and IHC	Cells	No	No	SHED has high angiogenic potential that hypoxia further increases
10.	Bento et al 71	SHED	*In vivo*	Mice	VEGF	PCR	Cells	EGM-2MV supplemented with VEGF	No	Increased blood vessel formation
**Periodontal ligament–derived mesenchymal stem cells (PDLSCs)**										
1.	Iwasaki et al 72	PDL	*In vitro*	HUVECs	VEGF	ELISA	Conditioned media	No	HUVECs	HUVECs demonstrated minimal apoptotic activity on treatment with PDLSC-CM; increased vascular activity was noted at the same time
2.	Zhang et al 73	PDL	*In vitro*	HUVECs	CD31 and VEGFA	Flow cytometry	No	No	HUVECs	HUVECs treated with exosomes derived from inflamed PDLSCs exhibited better tube formation than the control group
3.	Diomede et al 74	PDL	*In vitro*	HUVECs	VEGF and RUNX2	Immunofluorescence and RT-PCR	Cells	Titanium surfaces, machined (CTRL) and dual acid-etched (TEST)	No	Human PDLSCs cultured on TEST evidenced a higher expression of VEGF and RUNX2 than hPDLSCs cultured on the CTRL surface
4.	Marconi et al 75	PDL	*In vitro*	HUVECs	VEGF, VEGF-R, and RUNX2	Immunofluorescence	Cells	Titanium implant surfaces modified with two different procedures, sandblasted (control—CTRL) and sandblasted/etched (test—TEST), as experimental titanium surfaces	No	TEST surfaces compared to CTRL titanium surfaces enhanced cell adhesion and increased VEGF and RUNX2 expression
5.	Kim et al 76	PDL	*In vitro*	HUVECs	VEGF, bFGF, and ANGPT1	PCR and Western blot analysis	Both	Cyclosporine A (CsA)	HUVECs	CsA reduced angiogenesis by blocking the ERK and p38/c-fos pathway in hPDLSCs
6.	Iwasaki et al 77	PDL	*In vivo*	Rat	VEGF, bFGF, and HGF	Flow cytometry and PCR	Cells	No	No	VEGF expression was increased in PDLSCs
7.	Jearanaiphaisarn et al 78	PDL	*In vitro*	HUVECs	VEGF, alpha-1 type I collagen (COL1), and essential bFGF	qPCR, ELISA, immunofluorescence staining	Cells	Iloprost, prostacyclin receptor (IP) antagonist	No	Iloprost promoted mRNA and protein expression of VEGF and COL1, but not of bFGF in hPDLSCs cells
8.	Wei et al 79	PDL	*In vitro*	HUVECs	bFGF and Ang	PCR and flow cytometry	Cells	No	PDLSCs from healthy teeth and periodontally compromised teeth, rapamycin, and cDNA-Beclin-1	Proangiogenic cytokine expression increased, and more tube formation was observed in periodontally compromised teeth derived PDLSCs
9.	Bae et al 80	PDL	*In vivo*	Mice	Stromal cell–derived factor 1 (SDF-1)	PCR and immunofluorescent	Cells	CXCR4 antagonist	HUVECs	Co-injection of PDLSCs and HUVECs worked up well for establishing vascular anastomosis
**Stem cells from apical papilla (SCAPs)**										
1.	Yi et al 81	SCAPs	*In vivo*	Mice	CD31, VEGFR2, VEGFR1, and TIE2	RT-PCR, western blotting, flow cytometry, and immunofluorescence	Cells	Acetylated low-density lipoprotein (ac-LDL)	HUVECs, SCAPs-endothelial cells	Angiogenic factors enhanced the differentiation of SCAPs into endothelial cells
2.	Liu et al 82	SCAPs	*In vitro*	Endothelial cell lines	Hypoxia-inducible factor-1α (HIF-1α) and VEGF	RT-PCR and ELISA	Both	Cobalt-doped multiwalled carbon nanotube nanocomposites	Endothelial cells	Conditioned media collected from SCAP when treated with nanocomposites showed enhanced vessel formation
3.	Yu et al 83	SCAPs	*In vitro*	HUVECs	VEGF and FGF-2	RT-PCR and immunofluorescence staining	Both	No	BM-MSCs, dental pulp cells (DPCs)	SCAPs-CM showed enhanced osteogenic and neurogenic differentiation in DPCs but did not prove to be significant in angiogenesis
4.	Yuan et al 84	SCAPs	*In vivo*	Mice	VEGF	PCR and ELISA	Cells	SCAPs transduced with an ephrinB2-lentiviral expression vector (ephrinB2-SCAPs) in the experimental group and green fluorescent protein (GFP-SCAPs) in the control group	HUVECs	Enhanced expression of VEGF was observed with ephrinB2 transduction
5.	Koutsoumparis et al 85	SCAPs	*In vitro*	HUVECs	PECAM-1, VEGFR2, vWF, and VE-cadherin/CDH5 MMP-2	RT-PCR and flow cytometric analysis	Cells	Recombinant human erythropoietin-alpha (rhEPOa)	No	rhEPOa is capable of promoting endothelial transdifferentiation of SCAP
6.	Yadlapati et al 86	SCAPs	*In vivo*	Mice	Left-right determination factor 1 (LEFTY1), bone morphogenetic protein 8b (BMP8B), peptidylprolyl isomerase A (PPIA), bone morphogenetic protein 4 (BMP4), TGFβ1, FGF5, colony-stimulating factor 1 (CSF1), VEGFC, pleiotrophin (PTN), and ubiquitin C (UBC), VEGFA, PPIA, chemokine (C-X-C motif) ligand 1 (CXCL1), hydroxymethylbilane synthase (HMBS), RPL0, and inhibin beta A (INHBA)	ELISA	Cells	VEGF loaded (concentration of 12.2 ng/cm) polydioxanone fiber	No	Accelerated angiogenesis was achieved
7.	Yuan et al 87	SCAPs	*In vitro*	HUVECs	VEGF	PCR and ELISA	Cells	EphrinB2	HUVECs	Co-culture of SCAPs and HUVECs accelerated the formation of vascularlike structures while inhibition of EphrinB2 expression suppressed the formation of vessel-like structures
8.	Peters et al 88	SCAPs	*In vitro*	HUVECs	VEGF, ANGPT1, c-fos0-induced growth factor (FIGF), FGF2, and TGFβ1	Flow cytometry and PCR	Cells	ProRoot MTA or BD	No	VEGF expression was enhanced by stimulating either MTA or BD types of cement, but FGF and ANGPT1 expression were reduced
9.	Bakopoulou et al 89	SCAPs	*In vitro*	HUVECs	Angiogenin, IGFBP-3, VEGF, PDGF, IGF1, MMPs, PECAM-1, and VE-cadherin	PCR, flow cytometry, and ELISA	Both	SCAP was exposed to serum deprivation (SD), glucose deprivation (GD), and oxygen deprivation/hypoxia (OD) conditions	HUVECs	Exposing the cells to stressed conditions proved to enhance the angiogenesis obtained from CM
10.	Yuan et al 90	SCAPs	*In vitro*	HUVECs	VEGF, EphrinB2, angiopoietin, EphB4, insulin growth factor-1, EDN1, FGF, PDGF, and TGF-β	ELISA and RT-PCR	Cells	Hypoxic conditions	HUVECs	HIF-1a and ephrinB2 in SCAP under hypoxia are upregulated
**Gingival mesenchymal stem cells (GMSCs)**										
1.	Jin et al 91	GMSCs	*In vivo*	Mice	VEGF-A, TGF-β, and FGF-2	ELISA and RT-PCR	Both	Lentivirus transfection and FGF-2	HUVECs	FGF-2 gene-modified GMSCs constructed using lentiviral transfection promoted GMSCs paracrine of angiogenesis-related growth factors
**Comparison of OC-MSC sources**										
1.	Zhu et al 92	SHED and DPSC	*In vitro*	HUVECs	PDGFR-β, α-SMA, NG2, and DEMSIN	RT-PCR	Cells	No	HUVECs	DPSCs performed better as a candidate in angiogenic assays compared to SHED
2.	Xie et al 93	SHED and DPMSC	*In ovo*	Chick embryo CAM	PECAM-1/CD31, VEGF, VEGF receptor 1 (VEGFR1), VEGF receptor 2 (VEGFR2), and vWF	RT-PCR	Cells	No	BM-MSCs	Angiogenic gene expressions were increased in SHED compared to DPMSCs or BM-MSCs
3.	Angelopoulos et al 94	Gingival MSCs (GMSCs) and DPMSCs	*In vivo*	Mice	VEGF and HGF	Flow cytometry, ELISA, and IHC	Both	No	No	GMSCs showed an improved angiogenic capacity compared to DPMSCs
4.	Xu et al 95	DPMSCs and SHED	*In vivo*	Mice	VEGF-A, VEGF-RI, PlGF-1, TGF-β, and SB-431542	RT-PCR and IHC	Cells	No	No	SHED possessed a higher endothelial differentiation potential than DPMSCs
5.	Osman et al 96	PDLSCs and SHED	*In vitro*	HUVECs	TGF, IGF, FGF, VEGF, PDGF, and CTGF	PCR	Cells	No	No	PDLSCs showed a higher propensity toward angiogenesis compared to DPMSCs
**Combined sources of stem cell**										
1.	Zhang et al 97	DPSCs and SHED	*In vivo*	Mice	VEGFR2, Tie-2, CD31, and VE-cadherin	Flow cytometry	Cells	No	No	p53/p21 regulates the angiogenic potential of DPSCs and SHED *in vivo*
2.	Olcay et al 98	DPMSCs, PDLSCs, and human tooth germ stem cells (hTGSCs)	*In vitro*	HUVECs	FGF-2, PDGF, and VEGF	Flow cytometry and ELISA	Both	Tricalcium silicate-based MRA (ProRoot MTA), BD, and a novel bioceramic root canal sealer (Well-Root ST) and Dycall are positive control groups	HUVECs	VEGF levels were significantly higher in a ProRoot MTA group
3.	Hilkens et al 99	DPMSCs and SCAPs	*In vivo*	Mice	VEGF, primary bFGF, angiopoietin-1, MMPs, endostatin, thrombospondin-1, and IGFBP3	ELISA and IHC	Cells	No	No	Co-culture of DPMSCs and SCAPs provided enhanced angiogenic proliferation of cells and improved blood vessel growth *in vivo*
4.	Zhang et al 100	DPMSCs and SHED	*In vivo*	Mice	VEGF, Wnt-β-catenin	PCR and IHC	Cells	No	No	Wnt/b-catenin silencing depressed angiogenesis by DPMSCs
5.	Hilkens et al 101	SCAPs and DPMSCs	*In ovo*	Chorioallantoic membrane	VEGF, bFGF, HGF-1, ANGPT1, and IGFBP3	PCR and ELISA	Both	No	No	DPMSCs and SCAPs caused a significant increase in blood vessel count

The following data items were extracted: authors and year of publication; source of stem cells used; type of study; model used for evaluating angiogenesis; growth factors assessed for angiogenesis; method used for analysis of angiogenesis; use of stem cells/conditioned media; use of preconditioning; use of co-culture with MSCs; and results obtained.

To evaluate an article's quality, we used the Joanna Briggs Institute appraisal checklist for a case-control study. Based on 10 prespecified questions in the tool, two researchers independently examined all case reports. Each question received one of the following statuses based on judgment: “yes,” “no,” “maybe,” or “unclear.” A quality grade was assigned to the listed studies, with scores over 70% deemed excellent. Scores between 40 and 70% were considered to be of moderate quality, while those under 40% were considered to be of low quality. The reviewers agreed on these criteria in order to provide a thorough and objective assessment of the research quality. Egger's regression test was used to identify publication bias in the selected articles for quantitative analysis.

## Results

**Fig. 1:**
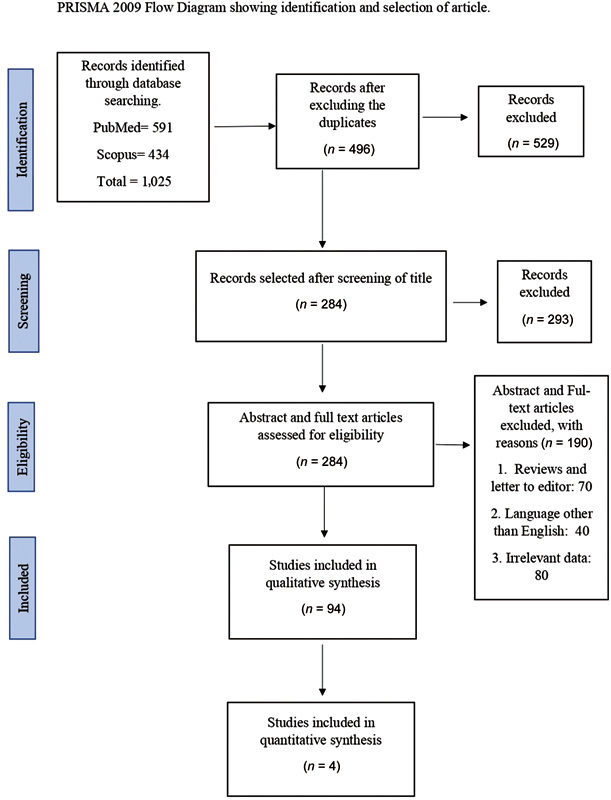
Preferred Reporting Items for Systematic Reviews and Meta-Analyses (PRISMA) chart illustrating the research methodology used in the review.

**Fig. 2:**
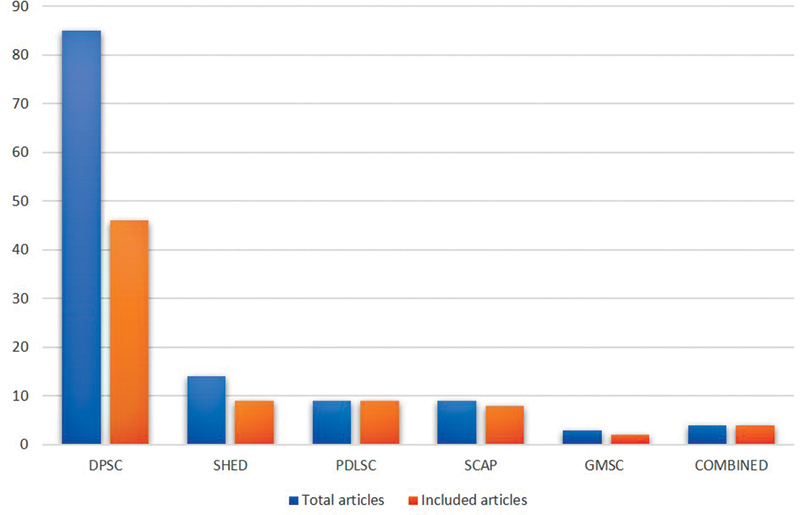
Graphical representation of the source-wise articles included in the review. DPSC, dental pulp stem cell; GMSC, gingival mesenchymal stem cell; PDLSC, periodontal ligament–derived mesenchymal stem cell; SCAP, stem cells from apical papilla; SHED, stem cells from human exfoliated deciduous teeth.

In an initial literature search, 1,025 articles (591 from PubMed and 434 from Scopus) were retrieved. The selection strategy employed in the qualitative and quantitative analysis is illustrated using the PRISMA flowchart. The results of database searches were carefully maintained using Mendeley software (version 1803). Mendeley software (version 1803) for Windows (Elsevier, London, UK) was used in the initial phase of the screening process to remove duplicate articles. Five hundred and twenty-nine articles from both databases were excluded due to overlapping data. After scrutiny of the titles, 284 articles were selected. Abstracts and full texts of the remaining articles were further screened for relevance, and 80 articles were excluded. In addition, 70 reviews and letters to editors were excluded. Of the remaining 134 articles, 40 were excluded due to data being in languages other than English or irrelevance. Hence, a total of 94 articles were selected for data extraction. The data extracted from the included studies are summarized in Table 1 . Fig. 1 summarizes and depicts the PRISMA flowchart. Source-wise number of articles included in the review are depicted in the graph in Fig. 2 .

**Fig. 3:**
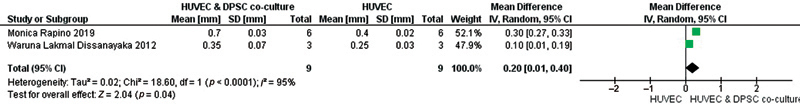
Summary of the meta-analysis assessing the effect of DPMSCs on the tubular length in an *in vitro* Matrigel assay showing a positive correlation of the co-culture of HUVEC and DPMSCs with the tubule length formation, which was statistically significant ( *p* = 0.04). CI, confidence interval; DPMSCs, dental pulp–derived mesenchymal stem cells; DPSC, dental pulp stem cell; HUVEC, human umbilical vein endothelial cell; SD, standard deviation.

**Fig. 4:**
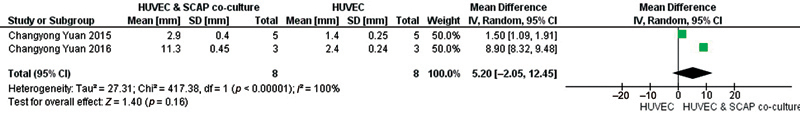
Summary of the meta-analysis assessing the effect of SCAP on the tubular length in an *in vitro* Matrigel assay showing a positive correlation of the co-culture of HUVEC and SCAPs with the tubule length formation, which was not statistically significant ( *p* = 0.16). CI, confidence interval; HUVEC, human umbilical vein endothelial cell; SCAP, stem cells from apical papilla; SD, standard deviation.

**Fig. 5:**
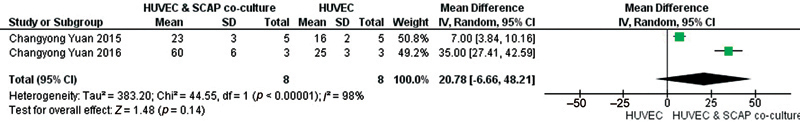
Summary of the meta-analysis assessing the effect of SCAP on the total branching points in an *in vitro* Matrigel assay showing a positive correlation of the co-culture of HUVEC and SCAPs with the total branching point number, which was not statistically significant ( *p* = 0.14). CI, confidence interval; HUVEC, human umbilical vein endothelial cell; SCAP, stem cells from apical papilla; SD, standard deviation.

A quantitative analysis was possible only for comparing studies with an individual oral-derived source of MSCs. The DPMSCs and SCAPs were individually compared with the endothelial cell lines used to control *in vitro* analysis of blood vessel formation. Thus, the best source of oral-derived MSCs is not projected through the meta-analysis performed. Instead, the present meta-analysis shows that oral tissue–derived stem cells have more potential for augmenting angiogenesis than endothelial cell lines alone. The studies compared the *in vitro* tubule formation or total branching points between cases and controls. Out of the 94 studies in the systematic review, only four had data compatible with a meta-analysis. 34 61 84 90 These studies referred to tubular formation's mean and total branch points in the case and control groups. The difference between the mean with standard deviation and the corresponding confidence interval was calculated for each study. Forest plots were created with RevMan software (version 5.4.1) using the calculated mean differences shown in Figs. 3 , 4 , 5 .

The meta-analysis ( Fig. 3 ) shows a positive correlation of the co-culture of human umbilical vein endothelial cells (HUVECs) and DPMSCs with tubule length formation, which was statistically significant ( *p* = 0.04), with a mean difference of 0.20 and a 95% confidence interval of 0.01–0.40. Succeeding meta-analysis ( Figs. 4 and 5 ) showed a positive correlation with the co-culture of HUVEC and the SCAP group with tubule length formation ( Fig. 4 ) and total branching points ( Fig. 5 ) with a mean difference of 5.20 and 20.78 and a 95% confidence interval of –2.05 to 12.45 and –6.66 to 48.21, respectively. Thus, the overall results from the meta-analysis revealed that oral-derived MSCs (DPSC and SCAP) carry a better angiogenic potential *in vitro* than the endothelial cell lines used alone, as depicted in the forest plot in Figs. 3 , 4 , 5 .

### Assessment of Quality and Publication Bias

Ten of the 94 studies considered obtained ratings less than 70%, categorizing them as intermediate in quality. In contrast, the other 84 studies were classed as high quality given that their overall score surpassed 70%. The studies included for meta-analysis were high quality with score greater than 70%.

**Fig. 6:**
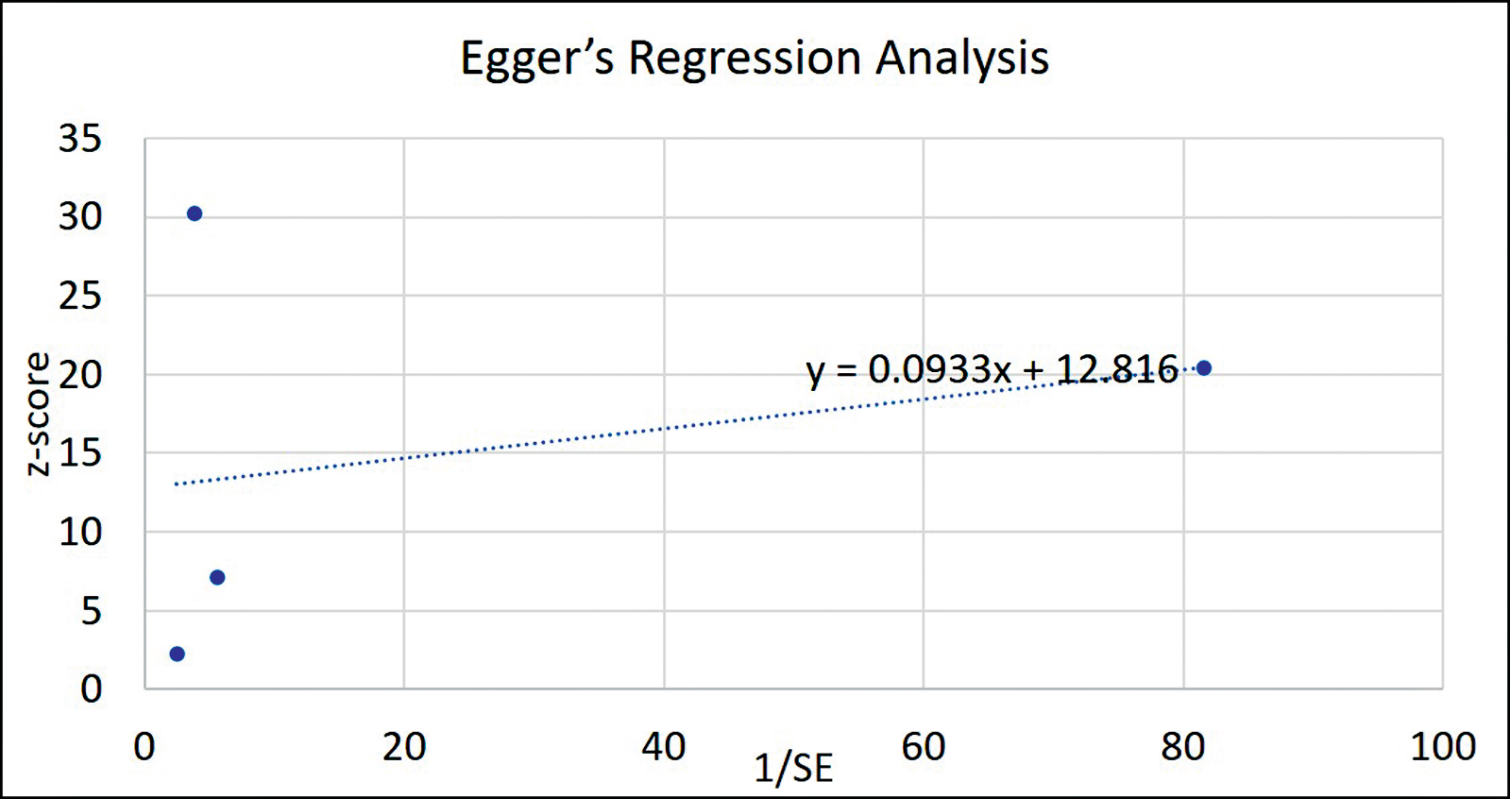
Graphical representation of Egger's regression test.

The Egger test showed a potential publication bias with 50% studies closer to the intercept line and 50% of studies away from the intercept line ( Table 2 , Fig. 6 ). Such skewed results could be attributed to small sample of studies that were analyzed quantitatively.

**Table 2:** Tabular representation of Egger's regression test

**Study reference**	***Z*** **-score**	**SD**	***n***	**SE**	**1/SE**
2012	2.27	0.7	3	0.404	2.474
2015	7.11	0.4	5	0.179	5.590
2016	30.23	0.45	3	0.260	3.849
2019	20.38	0.03	6	0.012	81.650

Abbreviations: SD, standard deviation; SE, standard error.

## Discussion

After a detailed scrutiny of the literature, 94 articles meeting our inclusion criteria were included in the review, investigating the influence of MSCs or their secretomes derived from oral sources. Of these, 54 studies involved dental pulp, 10 articles investigated MSCs from SHED, and 9 investigated the PDL stem cells. The SCAPs were studied in 10 articles, and gingival MSCs (GMSCs) were explored in a single study. DPMSCs were relatively more explored for their angiogenic potential, as evidenced by the number of articles published. The critical parameters investigated to assess the effect of OC-MSCs and their secretomes on angiogenesis were tube capillary length and diameter, branching points, number of loops, expression of angiogenic proteins, endothelial cell proliferation in *in vitro* studies and capillary formation, enhanced wound healing, and generation of neovascularization in *in ovo* and *in vivo* studies. Postnatal MSCs (DPMSCs, PDL-derived stem cells [PDLSCs], SHED, GMSCs, and SCAP) retain the unique ability to form new functional blood vessels through angiogenesis. 97

### Dental Pulp–Derived Mesenchymal Stem Cells

The dental pulp is a rich source of MSCs that exhibit a self-renewal multilineage differentiation potential and secrete multiple proangiogenic factors. Thus, among the several therapeutic applications under investigation, the ability of DPMSCs to enhance angiogenesis has been the subject of active investigation.

Interestingly, the co-culture of DPMSCs with HUVECs exhibited a thick vessel-like structure, a characteristic feature of angiogenesis. The formation of vessel-like structures was absent in untreated HUVECs, confirming the angiogenic role of DPMSCs. 55 61 DPMSCs could induce angiogenesis in a chicken chorioallantoic membrane model, as shown by the increased capillaries that observe a typical spoke wheel pattern around the DPMSCs Matrigel. 58 DPMSCs mediated noticeable repair of the infarcted myocardium in the animal model of myocardial infarction as an increase in the total number of blood vessels and an overall reduction in the infarct size was apparent. Therefore, the authors suggested DPMSCs as a potential alternative to bone marrow–derived MSCs to treat myocardial infarction. 59 102 DPMSC-derived cells could promote neovasculogenesis in the mouse brain. 39

Secretomes derived from DPMSCs have been actively investigated for their proangiogenic role. DPMSC secretomes also potentially enhance the proliferation of HUVECs. 24 DPMSC secretomes promoted angiogenesis in endothelial cell progenitors and terminally differentiated endothelial cells, as evidenced by the formation of tubelike structures in the Matrigel assay. In addition, DPMSC secretomes have been shown to improve the capillary density of skeletal muscles through improved angiogenesis, which can be attributed to the VEGF content in the secretomes. In the transwell migration assay performed on HUVECs, DPMSC secretomes promote better migration of HUVECs and microvascular network formation than the endothelial growth medium (EGM), suggesting a profound angiogenic role of DPMSC secretomes. 42 Under serum-free conditions, DPMSC secretomes have been shown to enhance the capillary tubelike formation from preexisting blood vessels, ultimately assisting angiogenesis. 29

In a co-culture of secretomes derived from DPMSCs and bone marrow–derived MSCs, substantial proangiogenic changes were observed in the chorioallantoic membrane. 22 Furthermore, local intramuscular injection of DPMSC secretomes in the hindlimb ischemic mice model showed enhanced neovascularization and marked improved blood perfusion at the ischemic site. 54 60 62 Similar results were found in a mice model of ectopic tooth transplantation wherein enhanced expression of VEGF was noted, promoting pulp regeneration. 30 77 Furthermore, DPMSC secretomes could promote pulplike vascularization in a scaffold implanted in a mouse model. 45

One of the added therapeutic benefits of MSCs is their ability to secrete EV containing various nucleic acids, lipids, and proteins into the extracellular space. Many studies have suggested that EVs from MSCs can be employed for therapeutic applications in recent times. Interestingly, fibrin gel loaded with DPMSC-derived EVs enhanced cell migration and vascular tube formation in *in vitro* culture. 27 A mouse model was used to assess wound healing over the skin, where EVs derived from DPMSCs of healthy and periodontally compromised teeth were included. The results showed that EVs from DPMSCs from periodontally compromised teeth (P-DPMSCs) accelerated wound healing in mice compared to those derived from DPMSCs from healthy teeth.

Moreover, it showed enhanced blood vessel formation/angiogenesis, which forms the basis of wound healing, suggesting that the inflammatory microenvironment enhances the proangiogenic effects of DPMSCs. A comparative analysis between the DPMSCs derived from regular and deep carious teeth revealed that the expression levels of angiogenesis markers (VEGF, PDGF, stromal cell–derived growth factor-1) were higher in MSCs derived from deep carious pulp compared to the MSCs of the normal pulp. This suggests that an inflammatory microenvironment would instead work well for cell proliferation and further angiogenesis. 25 A combination of VEGF and IGF-1 enhances the angiogenic proliferation of DPMSCs from the carious environment synergistic effect. 32 Chronic inflammation-mediated tumor necrosis factor alpha induced initial apoptosis emerges DPSC into an angiogenic phenotype. 40 56 The role of DPMSC EVs in angiogenesis is evident as miR-424 plays a regulatory role in angiogenesis. 57 Recently, modulation of the proangiogenic potential of DPMSCs by preconditioning, altering the culture conditions, and using novel biomaterials yielded promising results. Hypoxic preconditioning could enhance the proangiogenic capacity of DPMSCs. 43 53 The expression of HIF-1α and SENP1 formed a positive feedback loop in angiogenesis promoted by DPMSCs under hypoxic conditions. HUVECs cultured with DPMSC secretomes treated with baicalein, 51 calcium phosphate cement (CPC), and CPC-bioactive glass nanoparticles (CPC-BGNs), 50 insulinlike growth factor binding protein 5 (IGFBP5) 10 exhibited higher expression of angiogenic markers in DPMSCs. DPMSCs treated with mineral trioxide aggregate (MTA), calcium hydroxide (Ca [OH]2), Biodentine (BD) and Emdogain, 23 EphrinB2-Fc, or EphB4-Fc 37 enhanced the expression of VEGF, which plays a crucial role in angiogenesis. 52

In contrast, treatment with triethylene glycol dimethacrylate (TEGDMA) alone at a concentration of 0.25 mM downregulated the expression of angiogenic factors, 38 clindamycin and minocycline 35 ; complete endothelial medium 2 (EGM-2) improved vessel formation; and angiogenic cell differentiation was achieved. 36 Aksel and Huang observed similar findings. 47 Treatment with 20% human platelet lysate under lipopolysaccharide-induced inflammatory environment in DPMSCs showed increased expression of proangiogenic markers. 6 Furthermore, the concentrated growth factor scaffold potentially enhanced endothelial cell proliferation and migration for DPMSCs. 41 Lipoprotein receptor–related protein signaling is required to express VEGF-promoting angiogenesis. 46 Decellularized matrix hydrogel derived from human dental pulp effectively promoted DPMSCs in a multidirectional differentiation. 31

### Stem Cells Obtained from Exfoliated Deciduous Teeth

SHED is a potent source of MSCs due to their higher proliferation potential, plasticity, and unique secretory profile. Few studies have explored the ability of SHED to enhance angiogenesis. Co-culture of the SHED with HUVECs promoted increased angiogenesis. 68 Furthermore, the SHED-HDMEC co-culture enhanced proangiogenic factor expression via NF-κB-dependent pathways. 65 Interestingly, SHED was subjected to shear stress-induced arterial endothelial differentiation. 4 SHED supplemented with an EGM showed augmented angiogenesis *in vivo* . 71 When subjected to a hypoxic environment, SHED augmented angiogenesis with improved function. 70 These studies suggest that SHED can be used as a perivascular source to form functional vascularlike structures *in vivo* . 76

### Periodontal Ligament–Derived Stem Cells

The PDL contains a population of progenitor cells, recently recognized as PDLSCs, capable of multilineage differentiation to produce tissues rich in collagen type I. Coadministration of PDLSCs and HUVECs showed anastomosis and enhanced blood vessel formation. It was seen that CXCR4 (an alpha-chemokine receptor specific for stromal-derived factor 1) antagonist inhibited blood vessel formation. This explains the role of PDLSCs in augmenting angiogenesis and blood vessel formation. 80 Furthermore, PDLSCs seeded on machined titanium disk surfaces showed increased VEGF expression, and RUNX2 (a gene inducing pluripotent stem cell differentiation to immature osteoblasts) plays a potential role in exhibiting angiogenesis. 74 In contrast, cyclosporine A–treated MSCs derived from PDL negatively impacted angiogenesis. 76

Furthermore, prostacyclin pretreated PDL stem cells negatively impacted iloprost enhanced angiogenic marker expression. 78 PDLSCs derived from healthy and inflamed tissue (periodontally compromised teeth) were subjected to proliferation and angiogenesis. The results depicted that the inflammatory microenvironment provided better augmentation for angiogenesis, which agrees with the findings on DPMSCs. 73 79

### Stem Cells Derived from Apical Papilla

A unique population of SCAP of the growing tooth root tips with embryoniclike properties is readily accessible in dental clinical practice from extracted wisdom teeth. Exposure of SCAP to various stress microenvironments and their respective secretomes has promoted angiogenesis. 89 EphrinB2 (a transmembrane ligand of EphB receptor tyrosine kinases expressed explicitly in arteries) could stabilize the vessel-like structure generated by the co-culture of SCAPs and HUVECs *in vitro* . 87 Co-culture of HUVECs and SCAPs under hypoxic conditions promoted the formation of endothelial tubules and a blood capillary network, which was in agreement with those obtained by Nam et al. 49 VEGF-loaded fibers can be considered a viable option for stimulating SCAP angiogenesis and new histogenesis during the endodontic procedure. 86 EphrinB2-transduced SCAPs could express VEGF marker in numerous amounts compared to the control group; its co-culture with HUVECs showed enhanced blood vessel formation in a Matrigel plug assay. 84 Treatment of SCAP cells with recombinant human erythropoietin-alpha (rhEPOa) elicits a proangiogenesis program by activating the Erythropoetin Receptor pathway. 85 Exposure of SCAP to MTA and BD (root-end filling material used in endodontic therapy of root canals) stimulated angiogenic gene expression and VEGF release inducing similar expression patterns in both MTA and BD. However, they appear to inhibit the expression of specific genes, including ANGPT1 and FGF2. 88 SCAP-derived secretomes improved osteogenic and neurogenic differentiation of dental pulp cells, but angiogenic differentiation did not significantly improve. 83

### Stem Cells Derived from Gingiva

The gingiva of human dentition is blessed with a remarkable contribution of neural crest ectomesenchyme, perifollicular mesenchyme, and partly the dental follicle proper. The origin of this tissue and its close approximation with the tooth give the GMSCs an exclusive position to stand apart from the rest of the oral cavity–derived cells. A study by Jin et al showed that when GMSCs were transfected with FGF-2, their expression potential for VEGF and TGF-β increased. Also, the secretomes derived from untreated GMSCs enhanced the gene and protein expression of angiogenic-related factors, endothelial tube formation, and cell migration capacity. However, the results obtained had an inferior efficacy than those obtained by the transfected GMSCs and their secretomes. 91

Several researchers have investigated the comparative potential of OC-MSCs to explore the ideal source of MSCs in the augmentation of angiogenesis. In a study by Angelopoulos et al, GMSCs potentially proliferate, migrate, and form angiogenic tubules better than DPMSCs *in vitro* and *in vivo* . 94 Another study performed by Xu et al compared SHED and DPMSCs in enhancing angiogenesis. Their findings revealed that SHED possesses better angiogenic potential than the DPMSCs. 95 Furthermore, SHED showed a more substantial angiogenesis differentiation and proliferation potential than DPMSCs. Furthermore, PDLSCs exhibited better angiogenic potential than DPMSCs. 96 However, very few studies have reported the comparative potential of OC-MSCs.

In yet another study, a co-culture of DPMSCs and SCAPs exhibited improved blood vessel formation *in vivo* . 99 Furthermore, in an *in ovo* angiogenesis assay, the co-culture of DPMSCs and SCAPs showed better angiogenesis than the single source. 101 A root canal obturating material, Well-Root ST stimulated neovascularization during endodontic regeneration procedures. Furthermore, Well-Root ST showed better efficacy than BD or ProRoot MTA for stimulation in various oral-derived MSCs (DPMSCs, SHED, PDLSCs, GMSCs, and SCAP). 98

The field of oral cavity–derived stem cells, particularly MSCs from dental pulp and apical papilla, has garnered interest due to their unique characteristics and potential applications in regenerative medicine. The finding that these stem cells have strong angiogenic potential holds several clinical implications and suggests promising directions for future research that could benefit the population in various ways.

- *Tissue regeneration:* The angiogenic potential of oral cavity–derived stem cells suggests their capability to stimulate the formation of new blood vessels. This can be extremely valuable in regenerating damaged tissues, such as those affected by injury, disease, or degeneration. These stem cells could aid in promoting blood supply and nutrients to the regenerating tissue, enhancing the overall healing process.
- *Wound healing:* The ability of these stem cells to promote angiogenesis can significantly accelerate wound healing in various clinical scenarios. For instance, they could be employed in chronic wound management, diabetic ulcer treatment, and postsurgical wound healing to expedite tissue repair and reduce complications.
- *Bone regeneration:* Oral-derived MSCs have shown potential for bone tissue regeneration. Enhancing angiogenesis could aid in developing more effective treatments for bone defects, fractures, and conditions like osteoporosis.
- *Dental applications:* The dental pulp and apical papilla are easily accessible sources of MSCs. This accessibility could make these stem cells valuable for various dental applications, such as periodontal tissue regeneration, dental implant support, and treatment of oral diseases.
- *Cardiovascular disorders:* Given their angiogenic properties, these stem cells might hold promise in treating cardiovascular diseases. They could stimulate the growth of new blood vessels in ischemic heart tissue, potentially reducing the impact of heart attacks.

## Limitations

The current literature shows a paucity of studies involving sources other than dental pulp. Even though OC-MSCs have proved their enhanced potential compared to other MSCs, further target-oriented comprehensive research is required to conclude which oral-derived stem cells have the most significant angiogenic potential. The systematic review involves different oral sources for MSCs, where maximum studies include dental pulp, and data for other sources (SHED, PDLSC, SCAP, and GMSC) are limited; therefore, a comparative evaluation could not be done. This systematic review incorporates *in vitro* , *ex vivo* , and *in vivo* trials and the data appear to be skewed. One specific type of research design might be advocated for better outcomes.

## Conclusion

The specific objectives of our study were to explore whether easily accessible OC-MSCs from dental pulp and apical papilla had good angiogenic potential. The reviewed literature shows that all the OC-MSCs augmented angiogenesis in various experiments. In the studies comparing DPMSCs and PDLSC, GMSCs, or SHED, the latter sources have shown increased significant potential for angiogenesis compared to that of the DPMSCs. MSCs obtained from different places show close phenotypic characteristics. However, it is still unclear how similar they are since proliferation and differentiation capabilities in the presence of different growth factor stimuli differ depending on the source of origin. For instance, bone marrow MSCs tend to lose their proliferative potential with age. DPSCs, on the other hand, have a higher proliferation index and growth potential. DPSCs show the highest odontogenic capability under the same inductive microenvironment in comparison to bone marrow stromal stem cells. 103

Avenues that can be explored further in the research realm are angiogenesis mechanisms, optimal delivery methods, combination therapy, and personalized medicine. This knowledge of precise molecular and cellular mechanisms underlying the angiogenic potential of oral-derived MSCs could lead to the development of targeted therapies. Future research could focus on identifying the most effective methods for delivering oral-derived MSCs to target tissues. This could involve investigating various delivery vehicles, such as scaffolds or hydrogels, to ensure the stem cells reach their intended destination. Furthermore, research might delve into tailoring treatments based on individual patient characteristics to maximize the regenerative potential. Exploring combination therapies, such as coupling oral-derived MSCs with growth factors or other regenerative agents, could enhance their angiogenic potential and effectiveness in various applications. Regenerative medicine and stem cells will usher in a renaissance in therapy in the near future.

The manuscript has been checked with the Fi-index tool and obtained a scrore of 0.60 for the first author on September 3, 2023 according to the Scopus database. The Fi-index tool aims to ensure the quality of the reference list and limit autocitations. 104 105

## References

[1] Honnegowda T M Kumar P Udupa E G Kumar S Kumar U Rao P Role of angiogenesis and angiogenic factors in acute and chronic wound healing Plast Aesthet Res 2015 2 243 249

[2] Auerbach R Auerbach W Polakowski I Assays for angiogenesis: a review Pharmacol Ther 1991 51 01 1 11 1722898 10.1016/0163-7258(91)90038-n

[3] Adair T H Montani J P Angiogenesis San Rafael, CA Morgan & Claypool Life Sciences 2010 1 84 21452444

[4] Wang P Zhu S Yuan C Wang L Xu J Liu Z Shear stress promotes differentiation of stem cells from human exfoliated deciduous teeth into endothelial cells via the downstream pathway of VEGF-Notch signaling Int J Mol Med 2018 42 04 1827 1836 30015843 10.3892/ijmm.2018.3761 PMC6108868

[5] King A Balaji S Keswani S G Crombleholme T M The role of stem cells in wound angiogenesis Adv Wound Care (New Rochelle) 2014 3 10 614 625 25300298 10.1089/wound.2013.0497 PMC4183912

[6] Bindal P Gnanasegaran N Bindal U Angiogenic effect of platelet-rich concentrates on dental pulp stem cells in inflamed microenvironment Clin Oral Investig 2019 23 10 3821 3831 10.1007/s00784-019-02811-5 30687907

[7] Chakraborty S Ponrasu T Chandel S Dixit M Muthuvijayan V Reduced graphene oxide-loaded nanocomposite scaffolds for enhancing angiogenesis in tissue engineering applications R Soc Open Sci 2018 5 05 172017 29892387 10.1098/rsos.172017 PMC5990794

[8] Kerkis I Kerkis A Dozortsev D Isolation and characterization of a population of immature dental pulp stem cells expressing OCT-4 and other embryonic stem cell markers Cells Tissues Organs 2006 184(3–4):105 116 17409736 10.1159/000099617

[9] Miran S Mitsiadis T A Pagella P Innovative dental stem cell-based research approaches: the future of dentistry Stem Cells Int 2016 2016 7.231038E6 10.1155/2016/7231038 PMC5018320 27648076

[10] Li J Zhu Y Li N Upregulation of ETV2 expression promotes endothelial differentiation of human dental pulp stem cells Cell Transplant 2021 30 9.63689720978739E14 10.1177/0963689720978739 PMC7863555 33522307

[11] Boreak N Khayrat N MA Shami A O Metformin pre-conditioning enhances the angiogenic ability of the secretome of dental pulp stem cells Saudi Pharm J 2021 29 08 908 913 34408549 10.1016/j.jsps.2021.07.004 PMC8363104

[12] Li Y Zhang Y Wang H Dental pulp mesenchymal stem cells attenuate limb ischemia via promoting capillary proliferation and collateral development in a preclinical model Stem Cells Int 2021 2021 5.585255E6 10.1155/2021/5585255 PMC8427677 34512766

[13] Li M Wang Q Han Q Novel molecule Nell-1 promotes the angiogenic differentiation of dental pulp stem cells Front Physiol 2021 12 703593 34512380 10.3389/fphys.2021.703593 PMC8427597

[14] Alghutaimel H Yang X Drummond B Nazzal H Duggal M Raïf E Investigating the vascularization capacity of a decellularized dental pulp matrix seeded with human dental pulp stem cells: *in vitro* and preliminary *in vivo* evaluations Int Endod J 2021 54 08 1300 1316 33709438 10.1111/iej.13510

[15] Zhou H Li X Wu R X Periodontitis-compromised dental pulp stem cells secrete extracellular vesicles carrying miRNA-378a promote local angiogenesis by targeting Sufu to activate the Hedgehog/Gli1 signalling Cell Prolif 2021 54 05 e13026 33759282 10.1111/cpr.13026 PMC8088471

[16] Huang X Qiu W Pan Y Exosomes from LPS-stimulated hDPSCs activated the angiogenic potential of HUVECs *in vitro* Stem Cells Int 2021 2021 6.685307E6 10.1155/2021/6685307 PMC8062194 33936213

[17] Afami M E El Karim I About I Coulter S M Laverty G Lundy F T Ultrashort peptide hydrogels display antimicrobial activity and enhance angiogenic growth factor release by dental pulp stem/stromal cells Materials (Basel) 2021 14 09 2237 33925337 10.3390/ma14092237 PMC8123614

[18] Liao Z H Zhu H Q Chen Y Y The epigallocatechin gallate derivative Y _6_ inhibits human hepatocellular carcinoma by inhibiting angiogenesis in MAPK/ERK1/2 and PI3K/AKT/ HIF-1α/VEGF dependent pathways J Ethnopharmacol 2020 259 112852 32278759 10.1016/j.jep.2020.112852

[19] He Y Cao Y Xiang Y An evaluation of norspermidine on anti-fungal effect on mature *Candida albicans* biofilms and angiogenesis potential of dental pulp stem cells Front Bioeng Biotechnol 2020 8 948 32903416 10.3389/fbioe.2020.00948 PMC7434867

[20] Guo S Redenski I Landau S Szklanny A Merdler U Levenberg S Prevascularized scaffolds bearing human dental pulp stem cells for treating complete spinal cord injury Adv Healthc Mater 2020 9 20 e2000974 32902147 10.1002/adhm.202000974

[21] Luzuriaga J Irurzun J Irastorza I Unda F Ibarretxe G Pineda J R Vasculogenesis from human dental pulp stem cells grown in matrigel with fully defined serum-free culture media Biomedicines 2020 8 11 483 33182239 10.3390/biomedicines8110483 PMC7695282

[22] Merckx G Hosseinkhani B Kuypers S Angiogenic effects of human dental pulp and bone marrow-derived mesenchymal stromal cells and their extracellular vesicles Cells 2020 9 02 312 32012900 10.3390/cells9020312 PMC7072370

[23] Caseiro A R Santos Pedrosa S Ivanova G Mesenchymal Stem/ Stromal Cells metabolomic and bioactive factors profiles: a comparative analysis on the umbilical cord and dental pulp derived stem/stromal cells secretome PLoS One 2019 14 11 e0221378 31774816 10.1371/journal.pone.0221378 PMC6881058

[24] Makino E Nakamura N Miyabe M Conditioned media from dental pulp stem cells improved diabetic polyneuropathy through anti-inflammatory, neuroprotective and angiogenic actions: cell-free regenerative medicine for diabetic polyneuropathy J Diabetes Investig 2019 10 05 1199 1208 10.1111/jdi.13045 PMC6717901 30892819

[25] Chen Y Li X Wu J Lu W Xu W Wu B Dental pulp stem cells from human teeth with deep caries displayed an enhanced angiogenesis potential *in vitro* J Dent Sci 2021 16 01 318 326 33384815 10.1016/j.jds.2020.03.007 PMC7770258

[26] Li J Rao Z Zhao Y A decellularized matrix hydrogel derived from human dental pulp promotes dental pulp stem cell proliferation, migration, and induced multidirectional differentiation *in vitro* J Endod 2020 46 10 1438 1.447E8 32679242 10.1016/j.joen.2020.07.008

[27] Wang D Lyu Y Yang Y Schwann cell-derived EVs facilitate dental pulp regeneration through endogenous stem cell recruitment via SDF-1/CXCR4 axis Acta Biomater 2022 140 610 624 34852303 10.1016/j.actbio.2021.11.039

[28] Zhou J Sun C SENP1/HIF-1α axis works in angiogenesis of human dental pulp stem cells J Biochem Mol Toxicol 2020 34 03 e22436 31953908 10.1002/jbt.22436

[29] Qu C Brohlin M Kingham P J Kelk P Evaluation of growth, stemness, and angiogenic properties of dental pulp stem cells cultured in cGMP xeno-/serum-free medium Cell Tissue Res 2020 380 01 93 105 31889209 10.1007/s00441-019-03160-1

[30] Zhu L Dissanayaka W L Zhang C Dental pulp stem cells overexpressing stromal-derived factor-1α and vascular endothelial growth factor in dental pulp regeneration Clin Oral Investig 2019 23 05 2497 2509 10.1007/s00784-018-2699-0 30315421

[31] Li J Diao S Yang H Cao Y Du J Yang D IGFBP5 promotes angiogenic and neurogenic differentiation potential of dental pulp stem cells Dev Growth Differ 2019 61 09 457 465 31599466 10.1111/dgd.12632

[32] Lu W Xu W Li J Chen Y Pan Y Wu B Effects of vascular endothelial growth factor and insulin growth factor–1 on proliferation, migration, osteogenesis and vascularization of human carious dental pulp stem cells Mol Med Rep 2019 20 04 3924 3932 31485628 10.3892/mmr.2019.10606

[33] Youssef A R Emara R Taher M M Effects of mineral trioxide aggregate, calcium hydroxide, Biodentine and Emdogain on osteogenesis, Odontogenesis, angiogenesis and cell viability of dental pulp stem cells BMC Oral Health 2019 19 01 133 31266498 10.1186/s12903-019-0827-0 PMC6604301

[34] Rapino M Di Valerio V Zara S Chitlac-coated thermosets enhance osteogenesis and angiogenesis in a co-culture of dental pulp stem cells and endothelial cells Nanomaterials (Basel) 2019 9 07 928 31252684 10.3390/nano9070928 PMC6669739

[35] Dubey N Xu J Zhang Z Nör J E Bottino M C Comparative evaluation of the cytotoxic and angiogenic effects of minocycline and clindamycin: an *in vitro* study J Endod 2019 45 07 882 889 31133343 10.1016/j.joen.2019.04.007 PMC6612592

[36] Delle Monache S Martellucci S Clementi L *In vitro* conditioning determines the capacity of dental pulp stem cells to function as pericyte-like cells Stem Cells Dev 2019 28 10 695 706 30887879 10.1089/scd.2018.0192

[37] Gong T Xu J Heng B EphrinB2/EphB4 signaling regulates DPSCs to induce sprouting angiogenesis of endothelial cells J Dent Res 2019 98 07 803 812 31017515 10.1177/0022034519843886

[38] Schertl P Volk J Perduns R Impaired angiogenic differentiation of dental pulp stem cells during exposure to the resinous monomer triethylene glycol dimethacrylate Dent Mater 2019 35 01 144 155 30502225 10.1016/j.dental.2018.11.006

[39] Luzuriaga J Pastor-Alonso O Encinas J M Unda F Ibarretxe G Pineda J R Human dental pulp stem cells grown in neurogenic media differentiate into endothelial cells and promote neovasculogenesis in the mouse brain Front Physiol 2019 10 347 30984027 10.3389/fphys.2019.00347 PMC6447688

[40] Zou T Jiang S Dissanayaka W L Sema4D/PlexinB1 promotes endothelial differentiation of dental pulp stem cells via activation of AKT and ERK1/2 signaling J Cell Biochem 2019 120 08 13614 13624 30937968 10.1002/jcb.28635

[41] Jin R Song G Chai J Gou X Yuan G Chen Z Effects of concentrated growth factor on proliferation, migration, and differentiation of human dental pulp stem cells *in vitro* J Tissue Eng 2018 9 2.041731418817505E15 10.1177/2041731418817505 PMC6304703 30622693

[42] Gharaei M A Xue Y Mustafa K Lie S A Fristad I Human dental pulp stromal cell conditioned medium alters endothelial cell behavior Stem Cell Res Ther 2018 9 01 69 29562913 10.1186/s13287-018-0815-3 PMC5861606

[43] Dou L Yan Q Liang P Zhou P Zhang Y Ji P iTRAQ-based proteomic analysis exploring the influence of hypoxia on the proteome of dental pulp stem cells under 3D culture Proteomics 2018 18(3–4):1.700215E6 10.1002/pmic.201700215 29327447

[44] Aksel H Öztürk Ş Serper A Ulubayram K VEGF/BMP-2 loaded three-dimensional model for enhanced angiogenic and odontogenic potential of dental pulp stem cells Int Endod J 2018 51 04 420 430 29080346 10.1111/iej.12869

[45] Lambrichts I Driesen R B Dillen Y Dental pulp stem cells: their potential in reinnervation and angiogenesis by using scaffolds J Endod 2017 43(9S):S12 S16 28781091 10.1016/j.joen.2017.06.001

[46] Silva G O Zhang Z Cucco C Oh M Camargo C HR Nör J E Lipoprotein receptor–related protein 6 signaling is necessary for vasculogenic differentiation of human dental pulp stem cells J Endod 2017 43(9S):S25 S30 28778505 10.1016/j.joen.2017.06.006 PMC5657009

[47] Aksel H Huang G T Human and swine dental pulp stem cells form a vascularlike network after angiogenic differentiation in comparison with endothelial cells: a quantitative analysis J Endod 2017 43 04 588 595 28258811 10.1016/j.joen.2016.11.015 PMC5407702

[48] Zou T Dissanayaka W L Jiang S Semaphorin 4D enhances angiogenic potential and suppresses osteo-/odontogenic differentiation of human dental pulp stem cells J Endod 2017 43 02 297 305 28027822 10.1016/j.joen.2016.10.019

[49] Nam H Kim G H Bae Y K Angiogenic capacity of dental pulp stem cell regulated by SDF-1 α-CXCR4 axis Stem Cells Int 2017 2017 8.085462E6 10.1155/2017/8085462 PMC5447288 28588623

[50] Lee S I Lee E S El-Fiqi A Lee S Y Kim H W Stimulation of odontogenesis and angiogenesis via bioactive nanocomposite calcium phosphate cements through integrin and VEGF signaling pathways J Biomed Nanotechnol 2016 12 05 1048 1062 27305825 10.1166/jbn.2016.2209

[51] Lee S I Kim S Y Park K R Kim E C Baicalein promotes angiogenesis and odontoblastic differentiation via the BMP and Wnt pathways in human dental pulp cells Am J Chin Med 2016 44 07 1457 1472 27776430 10.1142/S0192415X16500816

[52] Spina A Montella R Liccardo D NZ-GMP approved serum improve hDPSC osteogenic commitment and increase angiogenic factor expression Front Physiol 2016 7 354 27594842 10.3389/fphys.2016.00354 PMC4990559

[53] Kuang R Zhang Z Jin X Nanofibrous spongy microspheres for the delivery of hypoxia-primed human dental pulp stem cells to regenerate vascularized dental pulp Acta Biomater 2016 33 225 234 26826529 10.1016/j.actbio.2016.01.032 PMC5975264

[54] Shen C Li L Feng T Dental pulp stem cells derived conditioned medium promotes angiogenesis in hindlimb ischemia J Tissue Eng Regen Med 2015 12 01 59 68

[55] Dissanayaka W L Zhu L Hargreaves K M Jin L Zhang C *In vitro* analysis of scaffold-free prevascularized microtissue spheroids containing human dental pulp cells and endothelial cells J Endod 2015 41 05 663 670 25687363 10.1016/j.joen.2014.12.017

[56] Boyle M Chun C Strojny C Chronic inflammation and angiogenic signaling axis impairs differentiation of dental-pulp stem cells PLoS One 2014 9 11 e113419 25427002 10.1371/journal.pone.0113419 PMC4245135

[57] Liu W Gong Q Ling J Zhang W Liu Z Quan J Role of miR-424 on angiogenic potential in human dental pulp cells J Endod 2014 40 01 76 82 24331995 10.1016/j.joen.2013.09.035

[58] Bronckaers A Hilkens P Fanton Y Angiogenic properties of human dental pulp stem cells PLoS One 2013 8 08 e71104 23951091 10.1371/journal.pone.0071104 PMC3737205

[59] Janebodin K Zeng Y Buranaphatthana W Ieronimakis N Reyes M VEGFR2-dependent angiogenic capacity of pericyte-like dental pulp stem cells J Dent Res 2013 92 06 524 531 23609159 10.1177/0022034513485599

[60] Ishizaka R Hayashi Y Iohara K Stimulation of angiogenesis, neurogenesis and regeneration by side population cells from dental pulp Biomaterials 2013 34 08 1888 1897 23245334 10.1016/j.biomaterials.2012.10.045

[61] Dissanayaka W L Zhan X Zhang C Hargreaves K M Jin L Tong E H Coculture of dental pulp stem cells with endothelial cells enhances osteo-/odontogenic and angiogenic potential *in vitro* J Endod 2012 38 04 454 463 22414829 10.1016/j.joen.2011.12.024

[62] Iohara K Zheng L Wake H A novel stem cell source for vasculogenesis in ischemia: subfraction of side population cells from dental pulp Stem Cells 2008 26 09 2408 2418 18583536 10.1634/stemcells.2008-0393

[63] Wu M Liu X Li Z SHED aggregate exosomes shuttled miR-26a promote angiogenesis in pulp regeneration via TGF-β/SMAD2/3 signalling Cell Prolif 2021 54 07 e13074 34101281 10.1111/cpr.13074 PMC8249784

[64] Han Y Chen Q Zhang L Dissanayaka W L Indispensable role of HIF-1α signaling in post-implantation survival and angio-/vasculogenic properties of SHED Front Cell Dev Biol 2021 9 655073 34368116 10.3389/fcell.2021.655073 PMC8343099

[65] Zaw S YM Kaneko T Zaw Z CT Crosstalk between dental pulp stem cells and endothelial cells augments angiogenic factor expression Oral Dis 2020 26 06 1275 1283 32248596 10.1111/odi.13341

[66] Atlas Y Gorin C Novais A Microvascular maturation by mesenchymal stem cells *in vitro* improves blood perfusion in implanted tissue constructs Biomaterials 2021 268 120594 33387754 10.1016/j.biomaterials.2020.120594

[67] Guo H Zhao W Liu A SHED promote angiogenesis in stem cell-mediated dental pulp regeneration Biochem Biophys Res Commun 2020 529 04 1158 1164 32819580 10.1016/j.bbrc.2020.06.151

[68] Gong T Heng B C Xu J Decellularized extracellular matrix of human umbilical vein endothelial cells promotes endothelial differentiation of stem cells from exfoliated deciduous teeth J Biomed Mater Res A 2017 105 04 1083 1093 28076902 10.1002/jbm.a.36003

[69] Kim J H Kim G H Kim J W *In vivo* angiogenic capacity of stem cells from human exfoliated deciduous teeth with human umbilical vein endothelial cells Mol Cells 2016 39 11 790 796 27871176 10.14348/molcells.2016.0131 PMC5125934

[70] Gorin C Rochefort G Y Bascetin R Priming dental pulp stem cells with fibroblast growth factor-2 increases angiogenesis of implanted tissue-engineered constructs through hepatocyte growth factor and vascular endothelial growth factor secretion Stem Cells Transl Med 2016 5 03 392 404 26798059 10.5966/sctm.2015-0166 PMC4807665

[71] Bento L W Zhang Z Imai A Endothelial differentiation of SHED requires MEK1/ERK signaling J Dent Res 2013 92 01 51 57 23114032 10.1177/0022034512466263 PMC3521451

[72] Iwasaki K Akazawa K Nagata M Angiogenic effects of secreted factors from periodontal ligament stem cells Dent J 2021 9 01 9 10.3390/dj9010009 PMC7829795 33467531

[73] Zhang Z Shuai Y Zhou F PDLSCs regulate angiogenesis of periodontal ligaments via VEGF transferred by exosomes in periodontitis Int J Med Sci 2020 17 05 558 567 32210705 10.7150/ijms.40918 PMC7085218

[74] Diomede F Marconi G D Cavalcanti M FXB VEGF/VEGF-R/RUNX2 upregulation in human periodontal ligament stem cells seeded on dual acid etched titanium disk Materials (Basel) 2020 13 03 706 32033260 10.3390/ma13030706 PMC7040902

[75] Marconi G D Diomede F Pizzicannella J Enhanced VEGF/VEGF-R and RUNX2 expression in human periodontal ligament stem cells cultured on sandblasted/etched titanium disk Front Cell Dev Biol 2020 8 315 32478069 10.3389/fcell.2020.00315 PMC7240029

[76] Kim H J Yoo J H Choi Y Joo J Y Lee J Y Kim H J Assessing the effects of cyclosporine A on the osteoblastogenesis, osteoclastogenesis, and angiogenesis mediated by human periodontal ligament stem cells J Periodontol 2020 91 06 836 848 31680236 10.1002/JPER.19-0168

[77] Iwasaki K Nagata M Akazawa K Watabe T Morita I Changes in characteristics of periodontal ligament stem cells in spheroid culture J Periodontal Res 2019 54 04 364 373 30597545 10.1111/jre.12637

[78] Jearanaiphaisarn T Sanharati T Pavasant P Nakalekha Limjeerajarus C The effect of iloprost on cell proliferation and angiogenesis-related gene expression in human periodontal ligament cells Odontology 2018 106 01 11 18 28547570 10.1007/s10266-017-0307-4

[79] Wei W An Y An Y Fei D Wang Q Activation of autophagy in periodontal ligament mesenchymal stem cells promotes angiogenesis in periodontitis J Periodontol 2018 89 06 718 727 29607508 10.1002/JPER.17-0341

[80] Bae Y K Kim G H Lee J C The significance of SDF-1α-CXCR4 axis in *in vivo* angiogenic ability of human periodontal ligament stem cells Mol Cells 2017 40 06 386 392 28614918 10.14348/molcells.2017.0004 PMC5523014

[81] Yi B Ding T Jiang S Conversion of stem cells from apical papilla into endothelial cells by small molecules and growth factors Stem Cell Res Ther 2021 12 01 266 33941255 10.1186/s13287-021-02350-5 PMC8091697

[82] Liu J Zou T Yao Q Zhang Y Zhao Y Zhang C Hypoxia-mimicking cobalt-doped multi-walled carbon nanotube nanocomposites enhance the angiogenic capacity of stem cells from apical papilla Mater Sci Eng C 2021 120 111797 10.1016/j.msec.2020.111797 33545919

[83] Yu S Zhao Y Fang T J Ge L Effect of the soluble factors released by dental apical papilla-derived stem cells on the osteo/odontogenic, angiogenic, and neurogenic differentiation of dental pulp cells Stem Cells Dev 2020 29 12 795 805 32178575 10.1089/scd.2019.0262

[84] Yuan C Wang P Zhu S Overexpression of ephrinB2 in stem cells from apical papilla accelerates angiogenesis Oral Dis 2019 25 03 848 859 30667136 10.1111/odi.13042

[85] Koutsoumparis A Vassili A Bakopoulou A Ziouta A Tsiftsoglou A S Erythropoietin (rhEPOa) promotes endothelial transdifferentiation of stem cells of the apical papilla (SCAP) Arch Oral Biol 2018 96 96 103 30205239 10.1016/j.archoralbio.2018.09.001

[86] Yadlapati M Biguetti C Cavalla F Characterization of a vascular endothelial growth factor–loaded bioresorbable delivery system for pulp regeneration J Endod 2017 43 01 77 83 27939739 10.1016/j.joen.2016.09.022

[87] Yuan C Wang P Zhu S EphrinB2 stabilizes vascularlike structures generated by endothelial cells and stem cells from apical papilla J Endod 2016 42 09 1362 1370 27451120 10.1016/j.joen.2016.05.012

[88] Peters O A Galicia J Arias A Tolar M Ng E Shin S J Effects of two calcium silicate cements on cell viability, angiogenic growth factor release and related gene expression in stem cells from the apical papilla Int Endod J 2016 49 12 1132 1140 26539648 10.1111/iej.12571

[89] Bakopoulou A Kritis A Andreadis D Angiogenic potential and secretome of human apical papilla mesenchymal stem cells in various stress microenvironments Stem Cells Dev 2015 24 21 2496 2512 26203919 10.1089/scd.2015.0197 PMC4620528

[90] Yuan C Wang P Zhu L Coculture of stem cells from apical papilla and human umbilical vein endothelial cell under hypoxia increases the formation of three-dimensional vessel-like structures *in vitro* Tissue Eng Part A 2015 21(5–6):1163 1172 25380198 10.1089/ten.tea.2014.0058 PMC4356259

[91] Jin S Yang C Huang J Conditioned medium derived from FGF-2-modified GMSCs enhances migration and angiogenesis of human umbilical vein endothelial cells Stem Cell Res Ther 2020 11 01 68 32070425 10.1186/s13287-020-1584-3 PMC7029497

[92] Zhu S Y Yuan C Y Lin Y F Stem cells from human exfoliated deciduous teeth (SHEDs) and dental pulp stem cells (DPSCs) display a similar profile with pericytes Stem Cells Int 2021 2021 8.859902E6 10.1155/2021/8859902 PMC8328701 34349804

[93] Xie J Zhao Y M Rao N Q Comparative study of differentiation potential of mesenchymal stem cells derived from orofacial system into vascular endothelial cells Beijing Da Xue Xue Bao Yi Xue Ban 2019 51 05 900 906 31624396 10.19723/j.issn.1671-167X.2019.05.018 PMC7433536

[94] Angelopoulos I Brizuela C Khoury M Gingival mesenchymal stem cells outperform haploidentical dental pulp-derived mesenchymal stem cells in proliferation rate, migration ability, and angiogenic potential Cell Transplant 2018 27 06 967 978 29770705 10.1177/0963689718759649 PMC6050910

[95] Xu J G Gong T Wang Y Y Inhibition of TGF-β signaling in SHED enhances endothelial differentiation J Dent Res 2018 97 02 218 225 28972822 10.1177/0022034517733741 PMC6429570

[96] Osman A Gnanasegaran N Govindasamy V Basal expression of growth-factor-associated genes in periodontal ligament stem cells reveals multiple distinctive pathways Int Endod J 2014 47 07 639 651 24182326 10.1111/iej.12200

[97] Zhang Z Oh M Sasaki J I Nör J E Inverse and reciprocal regulation of p53/p21 and Bmi-1 modulates vasculogenic differentiation of dental pulp stem cells Cell Death Dis 2021 12 07 644 34168122 10.1038/s41419-021-03925-z PMC8225874

[98] Olcay K Taşli P N Güven E P Effect of a novel bioceramic root canal sealer on the angiogenesis-enhancing potential of assorted human odontogenic stem cells compared with principal tricalcium silicate-based cements J Appl Oral Sci 2020 28 e20190215 31939521 10.1590/1678-7757-2019-0215 PMC6919198

[99] Hilkens P Bronckaers A Ratajczak J Gervois P Wolfs E Lambrichts I The angiogenic potential of DPSCs and SCAPs in an *in vivo* model of dental pulp regeneration Stem Cells Int 2017 2017 2.58208E6 10.1155/2017/2582080 PMC5605798 29018483

[100] Zhang Z Nör F Oh M Cucco C Shi S Nör J E Wnt/β-catenin signaling determines the vasculogenic fate of postnatal mesenchymal stem cells Stem Cells 2016 34 06 1576 1587 26866635 10.1002/stem.2334 PMC5338744

[101] Hilkens P Fanton Y Martens W Pro-angiogenic impact of dental stem cells *in vitro* and *in vivo* Stem Cell Res (Amst) 2014 12 03 778 790 10.1016/j.scr.2014.03.008 24747218

[102] Gandia C Armiñan A García-Verdugo J M Human dental pulp stem cells improve left ventricular function, induce angiogenesis, and reduce infarct size in rats with acute myocardial infarction Stem Cells 2008 26 03 638 645 18079433 10.1634/stemcells.2007-0484

[103] Zeidán-Chuliá F Noda M “Opening” the mesenchymal stem cell tool box Eur J Dent 2009 3 03 240 249 19756201 PMC2741198

[104] Fiorillo L Fi-index: a new method to evaluate authors Hirsch-index reliability Publ Res Q 2022 38 465 474

[105] Fiorillo L Cicciù M The use of Fi-index tool to assess per-manuscript self-citations Publ Res Q 2022 38 684 692

